# Comprehensive metabolic analyses provide new insights into primary and secondary metabolites in different tissues of Jianghua Kucha tea (*Camellia sinensis* var. *assamica* cv. Jianghua)

**DOI:** 10.3389/fnut.2023.1181135

**Published:** 2023-05-19

**Authors:** Wenliang Wu, Jiang Shi, Jiqiang Jin, Zhen Liu, Yong Yuan, Zhida Chen, Shuguang Zhang, Weidong Dai, Zhi Lin

**Affiliations:** ^1^Key Laboratory of Tea Biology and Resources Utilization, Ministry of Agriculture, Key Laboratory of Biology, Genetics and Breeding of Special Economic Animals and Plants, Ministry of Agriculture and Rural Affairs, Tea Research Institute, Chinese Academy of Agricultural Sciences, Hangzhou, Zhejiang, China; ^2^Tea Research Institute, Hunan Academy of Agricultural Sciences, Changsha, Hunan, China; ^3^Hunan Tea Group Co., Ltd., Changsha, Hunan, China; ^4^Chenzhou Guyanxiang Tea Co., Ltd., Chenzhou, Hunan, China

**Keywords:** Jianghua Kucha, leaf position, metabolomics, primary metabolites, secondary metabolites

## Abstract

**Background:**

Jianghua Kucha (JHKC) is a special tea germplasm with enriched specialized secondary metabolites, including theacrine, non-epimeric flavanols and methylated flavanols. Moreover, primary metabolites provide precursors and energy for the production of secondary metabolites. However, the accumulation patterns of primary and secondary metabolites in different tissues of JHKC are unclear.

**Methods:**

The changes of primary and secondary metabolites and related metabolic pathways (primary and secondary metabolism) in different JHKC tissues (the bud, 1st-4th leaves, and new stem) were investigated via metabolomics analysis with ultra-high-performance liquid chromatography quadrupole time-of-flight mass spectrometry (UHPLC-QTOF/MS).

**Results:**

Significant differences were observed in 68 primary and 51 secondary metabolites mainly related with the pathways of starch and sucrose, amino acids, caffeine, and flavanols metabolism and TCA cycle. The bud exhibited higher levels of glucose-6-phosphate, citric acid, most amino acids, theobromine, catechin-gallate, epicatechin-gallate, procyanidins, and theasinensins; the 1st leaf showed higher levels of caffeine and epigallocatechin-3-gallate; and the 4th leaf contained higher levels of most monosaccharides, theacrine, and epigallocatechin-3-O-(3”-O-methyl)-gallate. In addition, primary metabolites and important secondary metabolites had certain correlations.

**Conclusion:**

This study provides comprehensive insight into primary and secondary metabolites in JHKC and offers guidelines for efficiently utilizing specialized metabolites of JHKC in the future.

## 1. Introduction

Tea (*Camellia sinensis*) is among the most popular and oldest non-alcoholic beverages in the world and has attracted widespread attention for its health benefits. Kucha (*Camellia sinensis*) is a unique tea germplasm in China that has much bitterer taste than that of common tea cultivars. Jianghua Kucha [JHKC, *Camellia sinensis* var. *assamica* (CSA) cv. Jianghua] is a kind of Kucha and grows in the Nanling Mountain region in Hunan Province, southeastern China.

Our previous study (1) demonstrated that JHKC contains a special purine alkaloid (theacrine), and compared to common tea cultivars, such as Fudingdabai, Yunkang 10, and Zhuyeqi, JHKC shows significantly higher levels of non-epi-form flavanols including catechin (C), gallocatechin (GC), catechin gallate (CG), and gallocatechin gallate (GCG), as well as a methylated flavanol, epigallocatechin-3-O-(3″-O-methyl)-gallate (EGCG3″Me). Locals enjoy drinking Kucha tea to promote health, and some evidences (2, 3) have indicated that Kucha tea extract exhibits strong antioxidant and lipid-lowering properties. As an important special metabolite in JHKC, theacrine provides several benefits; for example, theacrine exhibits anti-depressive and anti-inflammatory activities and improves sleep and metabolism (4). GCG is the most abundant flavanol in JHKC except EGCG (1), and compared to EGCG, GCG exhibits stronger neuroprotective effects, antioxidant activity, ability to inhibit cholesterol biosynthesis, and antidiabetic activity (5–8). In addition, EGCG3″Me was reported to show stronger anti-allergic and anti-hypertensive effects than that of EGCG (9). The health value of tea is mainly associated with the composition of bioactive secondary metabolites (10); therefore, JHKC is a valuable tea germplasm.

The contents of secondary metabolites in tea trees vary among tissues, and compared with old tea shoots, new tea shoots are richer in secondary metabolites. However, reports on the distributions of secondary metabolites in different tissues were sometimes inconsistent, possibly due to different tea varieties (11, 12). In addition, most of these studies focused on the changes of secondary metabolites related to miRNA and protein expression in different tissues (12–15), whereas changes in primary metabolites were rarely reported. It is well known that primary metabolism provides precursors and energy for the production of secondary metabolites in plants (16); therefore, understanding the relationships between primary and secondary metabolites is essential for regulating important specialized metabolites. In particular, the distribution patterns of specialized metabolites (e.g., theacrine and flavanols) in different tissues of JHKC are largely unknown. Here, we investigated the changes of primary and secondary metabolites and related metabolic pathways (primary and secondary metabolism) in different JHKC tissues *via* metabolomics analysis with ultra-high-performance liquid chromatography quadrupole time-of-flight mass spectrometry (UHPLC-QTOF/MS). Our study provides a comprehensive analysis of important specialized metabolites in different tissues of JHKC tea plants and will be helpful for guiding the harvesting and manufacturing of JHKC tea leaves.

## 2. Materials and methods

### 2.1. Chemicals

Fifty-eight authentic standards of primary metabolites and 35 authentic standards of secondary metabolites were purchased from Sigma (St. Louis, MO, USA), Yuanye (Shanghai, China), ChemFaces (Wuhan, Hubei, China), J&K Scientific Ltd. (Beijing, China), and Nagara Science Co., Ltd. (Gifu, Japan) (the purchase information is shown in detail in the Material). Formic acid, methanol, ammonia, ammonium acetate, and acetonitrile (LC–MS grade) were purchased from Merck (Darmstadt, Germany). Deionized ultrapure water was obtained from Millipore (Bedford, MA, USA).

### 2.2. Plant materials

Five-year-old cloned tea plants, JHKC-37-2 (*Camellia sinensis* var. *assamica*), were grown in tea plantations of the Tea Research Institute (latitude N28.24, longitude E113.08), Hunan Academy of Agricultural Sciences, Changsha, Hunan, China. Samples of the bud, 1st-4th leaves, and new stem (no lignification) were harvested on April 11, 2020 (Supplementary Figure S1). These samples were collected with three biological replicates, and each biological replicate was collected from three randomly selected healthy tea trees. The samples were frozen in liquid nitrogen for metabolomics analysis.

### 2.3. Tea sample preparation

For primary metabolite analysis (17), 0.5 ml of a prechilled (−80°C) 80% (v/v) methanol solution was added to 50 mg of tea powder and then ground for 2 min on dry ice, after which the mixture was stored at −80°C overnight. The solution was centrifuged at 14,000 × *g* for 20 min at 4°C, and then the supernatant was transferred into a new tube. After being dried with nitrogen gas (room temperature), the samples were obtained and stored in a −80°C freezer pending analysis. Each tea sample was prepared in triplicate. Quality control (QC) samples were prepared by mixing a portion of every sample.

For secondary metabolite analysis (1), 100 mg tea powder was added to 15 ml of a prewarmed 70% methanol solution (v/v) and then incubated in a water bath at 70°C for 0.5 h. The tubes were cooled to room temperature and centrifuged at 8,000 × *g* for 15 min. After being filtered through a 0.22 μm membrane, the supernatants were obtained and stored in a −80°C freezer pending analysis. Each tea sample was prepared in triplicate. QC samples were prepared by mixing 100 μl aliquots of each tea infusion.

### 2.4. Metabolomics analysis

An Agilent 1,290 infinity system equipped with a 6,545 Q-TOF-MS system (Agilent Technologies, Santa Clara, CA, USA) was used to detect primary metabolites in both electrospray ionization ESI+ and ESI- modes. The separation was carried out on a BEH amide column (100 mm × 2.1 mm, 1.7 μm, Agilent Technologies, Little Falls, DE, USA) at 40°C column temperature. Mobile phase A was ultrapure water containing 0.3% ammonia and 15 mM ammonium acetate, and mobile phase B was acetonitrile/water (9:1) containing 0.3% ammonia and 15 mM ammonium acetate. The flow rate was 0.3 ml/min. The gradient of mobile phase A was held at 5% for 1 min, changed from 5 to 50% within 8 min, held at 50% for 3 min, changed from 50 to 10% within 0.5 min, and finally held at 5% for 6.5 min. The sample volume injected was 2 μl. The MS parameters used were as follows: capillary voltage, 3,500 V; nozzle voltage, 120 V; collision energy for MS^2^, 20 V; nebulizer gas, 35 psi; drying gas flow rate, 8 l/min; and gas temperature, 350°C. A full scan was run with a mass range from *m/z* 50 to 1,200. QC samples were injected after every nine samples to monitor the reproducibility of the metabolomic analysis results.

For secondary metabolite analysis, the LC–MS conditions on an UHPLC-Q-TOF/MS system were the same as those in our previous works (1, 18).

The raw metabolomics data analysis was conducted according to our previous procedure (1, 18) and is briefly described in the Supplementary material.

### 2.5. Absolute quantifications of important specialized metabolites

The absolute quantities of theanine, EGCG3”Me, eight flavanols (ECG, EGC, EGCG, EC, CG, GC, GCG, and C), and three alkaloids (theobromine, theacrine, and caffeine) were quantified by an UPLC-PDA-TQS system (Waters, Manchester, UK) using an external standard method according to our previous work (1).

### 2.6. Statistical and data analysis

Principal component analysis (PCA) and partial least squares discriminant analysis (PLS-DA) were performed using SIMCA 14.1 software (Umetrics, Umeå, Sweden). Statistical analyses of one-way ANOVA and Student’s *t* tests were performed using SPSS 21 software (IBM, Chicago, IL, USA). A heatmap was generated by MultiExperiment Viewer 4.9.0 (Oracle, Redwood, CA, USA) with UV scaling [(X- M)/SD; where X is the mass intensity in a sample, M is the average mass intensity in all samples, and SD is the standard deviation]. The correlation analysis between primary metabolites and important specialized secondary metabolites was conducted with a ggcorrplot package in R Project.^1^ The pathway analysis was performed by MetaboAnalyst 5.0^2^ on December 6th, 2022.

## 3. Results and discussion

### 3.1. Primary metabolite profiles among different tissues in JHKC accessions

**Figure 1:**
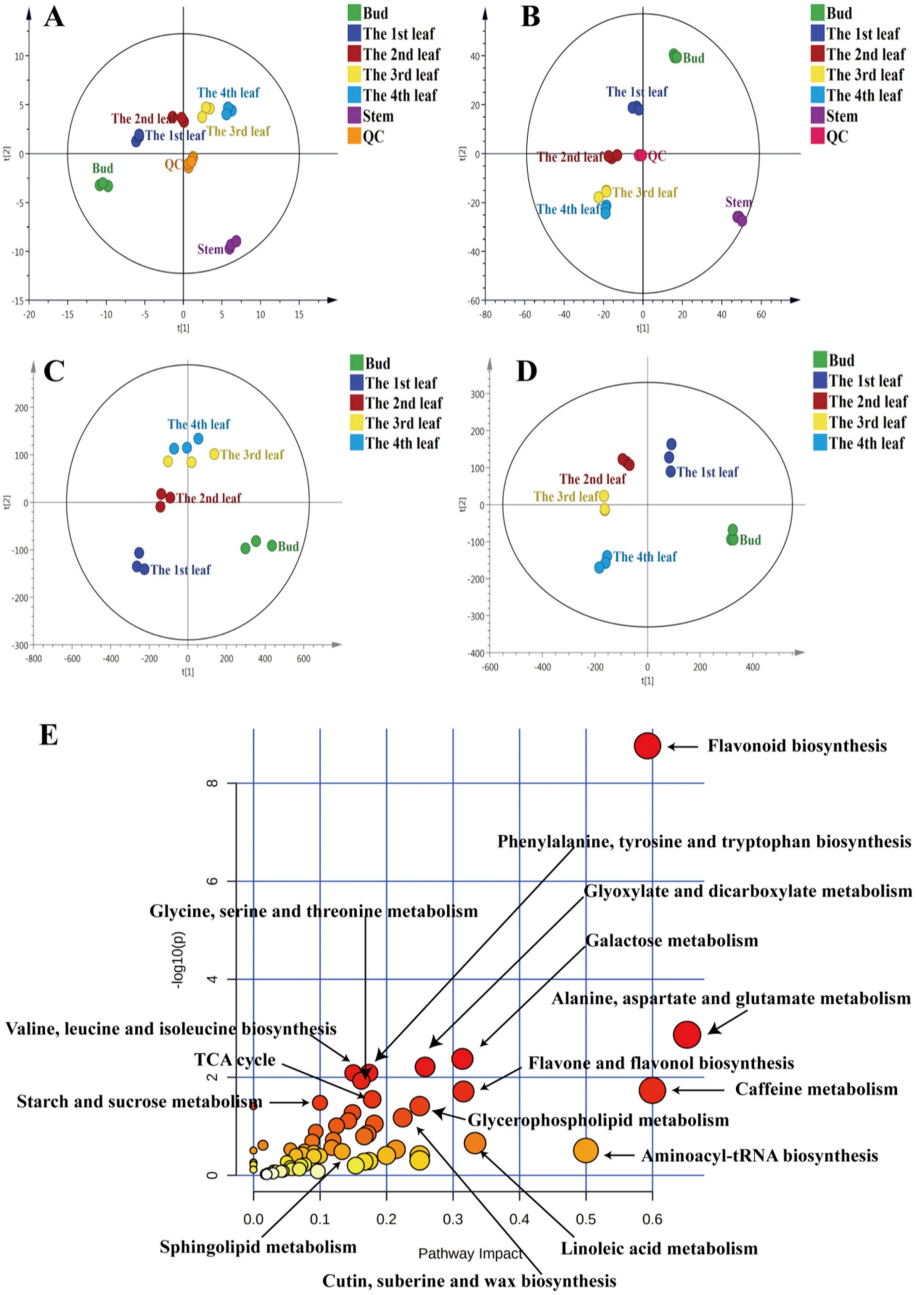
PCA of primary metabolites **(A)** and secondary metabolites **(B)**, PLS-DA of primary metabolites **(C)** and secondary metabolites **(D)**, metabolic pathway analysis of differential primary and secondary metabolites among JHKC tea leaves of different tissues **(E)**.

To understand the distribution of primary metabolites in different tissues of JHKC, the primary compounds were measured by LC–MS platform analysis. A PCA score plot was used to show the distribution of primary secondary metabolites in six tissues of JHKC (Figure 1A). The QC samples clustered at the center of the PCA score plot, indicating good reproducibility of the metabolomic analysis. The six tissue samples were well differentiated; in particular, the stem samples were far from the tea bud and leaf samples, which was in accordance with the hierarchical clustering analysis (HCA) results that the stem, bud and leaves were divided into two main categories (Supplementary Figure S2A). This likely occurred because the stem is rarely involved in photosynthesis and respiration processes. Then, the bud and the 1st-4th leaves were included for further PLS-DA analysis (Figure 1C). These samples showed distinct primary metabolite patterns in the PLS-DA score plot. A cross validation demonstrated that the *R^2^* and *Q^2^* intercepts were 0.7 and −0.47, respectively, indicating that the PLS-DA model was reliable (Supplementary Figure S2C). A total of 68 putatively identified primary metabolites, including 17 sugars/alcohols, 15 organic acids, 13 lipids, 20 amino acids, and three other compounds (Table 1), showed significant differences among the bud and the 1st-4th leaves (VIP > 1; *p* < 0.05, Duncan’s test).

**Table 1:** Summary of putatively identified primary metabolites in JHKC.

RT (min)	Accurate mass (m/z)	ESI Mode	Compound identification	MS^2^ fragments	*p*-value among 5 groups	VIP
Saccharides/Sugar alcohols						
5.62	343.1235	ESI+	Sucrose*	275, 224	0.022	4.64
6.04	180.0624	ESI-	Glucose*	59, 89, 70	0.016	3.24
6.72	261.0297	ESI+	Glucose-6-phosphate*	98, 109	0.014	5.11
6.95	166.9812	ESI-	Phosphoenolpyruvic acid	78, 148, 166	0.012	2.51
6.72	503.1675	ESI-	Raffinose*	503, 179, 221, 101	0.019	7.98
7.53	667.2292	ESI+	Stachyose*	145, 163, 505	0.012	2.69
6.11	343.1234	ESI+	Melibiose*	163, 145, 235	0.034	8
3.25	149.0456	ESI-	Ribose*	59	0.02	1.65
4.43	181.0706	ESI+	Mannose*	181, 162, 145, 163	0.008	3.23
4.9	179.0562	ESI-	Galactose*	59, 72	0.039	2.66
4.43	180.0872	ESI+	Glucosamine*	180, 162, 72	0.011	2.58
4.03	151.4526	ESI-	Xylitol*	71, 41, 43	0.013	2.05
6.45	181.0707	ESI+	myo-Inositol*	159, 138	0.035	3.03
4.86	181.0713	ESI-	Dulcitol*	181, 101, 71	0.01	2.36
5.29	196.0802	ESI+	Glucosamic acid*	131, 196, 179, 112	0.031	4.12
6.72	261.0296	ESI+	Galactose 1-phosphate	162	0.014	1.87
6.38	193.0348	ESI-	Galacturonic acid	72, 113, 85	0.009	1.75
Organic acids						
7.1	191.0193	ESI-	Citric acid*	111, 146, 162	0.016	2.23
4.02	191.0197	ESI-	Isocitric acid*	111, 116	0.009	3.01
5.85	145.0141	ESI-	α-ketoglutaric acid*	100, 57	0	6.11
6.12	117.0186	ESI-	Succinic acid*	73, 117	0.009	5.42
6.29	115.0033	ESI-	Fumaric acid*	71	0.001	4.41
5.21	115.0032	ESI-	Maleic acid*	71, 115	0.012	4.21
5.46	191.0554	ESI-	Quinic acid*	191, 85, 93	0.011	7.41
4.89	170.0225	ESI-	3-Dehydroshikimic acid*	127, 153, 109	0.021	2.79
5.28	173.0451	ESI-	Shikimic acid*	93, 111	0.04	2.12
5.77	160.0614	ESI-	2-Aminoadipic acid*	142, 160, 116, 98	0.009	2.66
3.43	138.0554	ESI+	4-Aminobenzoic acid*	138, 77, 94	0.021	8.15
5.82	166.0146	ESI-	Quinolinic acid*	122	0.012	4.39
5.16	130.0862	ESI+	L-Pipecolic acid*	84, 130	0.039	9.05
4.92	101.0237	ESI-	2-Ketobutyric acid*	101	0.036	5.36
5.94	290.1336	ESI+	Ophthalmic Acid*	58, 84	0.014	1.63
Lipids						
1.31	255.2326	ESI-	Palmitic acid*	255, 237	0.012	4.23
1.31	253.2176	ESI-	Palmitoleic acid*	253	0.037	1.25
1.29	283.2654	ESI-	Stearic acid*	283, 284	0.026	1.26
1.29	281.2485	ESI-	Oleic acid*	281	0.047	1.37
1.31	277.2173	ESI-	γ- Linolenic acid*	277, 260	0.01	5.12
1.28	312.3017	ESI-	Arachidic acid*	311, 100, 113	0.016	4.64
4.98	105.0193	ESI-	Glyceric acid	105, 75, 72	0.01	3.69
1.24	302.3057	ESI+	Sphinganine	206, 218, 332	0.011	2.55
1.27	337.3114	ESI-	Erucic acid	319, 213	0.012	2.65
1.34	758.5684	ESI+	Lecithin	/	0.032	6.32
6.71	173.0211	ESI+	Glycerol-3-phosphate	173, 75, 155	0.027	4.63
7	140.0114	ESI-	Phosphoethanolamine	78, 140	0.031	2.36
1.34	227.2021	ESI-	Myristic acid*	227, 228	0.023	5.32
Amino acids						
6.19	148.0604	ESI+	Glutamate	130, 148	0	2.36
5.65	147.0766	ESI+	Glutamine*	84, 130	0.009	5.18
5.72	156.0768	ESI+	Histidine*	110, 83, 93	0.009	3.24
4.96	116.0706	ESI+	Proline*	70, 116	0.012	2.21
7.52	175.1188	ESI+	Arginine*	70, 43	0.009	1.26
2.29	175.1079	ESI+	Theanine*	175, 130, 116, 70	0.001	1.56
5.78	104.071	ESI+	GABA*	158, 130, 84, 56	0.011	11.22
6.27	132.0305	ESI-	Aspartate*	86, 69	0	5.21
4.25	150.0584	ESI+	Methionine*	56, 104, 61, 133	0.001	2.56
5.69	385.1294	ESI+	S-adenosyl-L-homocysteine*	136, 88, 250, 385	0.012	6.21
5.35	120.0653	ESI+	Threonine*	74, 56	0.015	2.52
4.15	132.1019	ESI+	Isoleucine*	84, 74, 56	0.003	2.52
7.64	145.0979	ESI-	Lysine*	114, 86, 72, 56	0.026	1.68
3.76	205.0972	ESI+	Tryptophan*	146, 188, 144	0	6.32
4.6	182.0821	ESI+	Tyrosine*	188, 159, 146, 118	0.009	1.25
3.71	166.086	ESI+	Phenylalanine*	136, 119, 91, 77	0.017	8.71
4.25	118.0865	ESI+	Valine*	120, 103, 91, 77	0.021	5.21
3.88	130.0869	ESI-	Leucine*	72, 63, 58, 55	0.025	7.23
5.37	90.0551	ESI+	Alanine*	90, 55	0.009	2.96
5.71	106.0499	ESI+	Serine*	70, 88, 61	0.051	7.25
Others						
6.72	335.0636	ESI+	β-Nicotinamide mononucleotide*	123, 97	0.009	8.66
4.33	220.1181	ESI+	D-Pantothenic acid	90, 202	0.026	2.36
5.35	162.1125	ESI+	Carnitine*	162, 102	0.009	1.59

*The compound was identified by authentic compound (Metabolomics Standard Initiatives level 1); other compounds were identified by comparing their MS^2^ fragments with MassBank and HMDB databases (Metabolomics Standard Initiatives level 2); 5 groups were the bud and 1st-4th leaves.

Primary metabolites are necessary for every stage of growth and development in plants. Metabolic pathway analysis of the abovementioned 68 differential primary metabolites was performed by MetaboAnalyst 4.0 to investigate the differential metabolic pathways among the bud and 1st-4th leaves. The involved pathways mainly included alanine, aspartate and glutamate metabolism; aminoacyl-tRNA biosynthesis; linoleic acid metabolism; glyoxylate and dicarboxylate metabolism; glycerophospholipid metabolism; cutin, suberine, and wax biosynthesis; glycine, serine and threonine metabolism; valine, leucine and isoleucine biosynthesis; phenylalanine, tyrosine and tryptophan biosynthesis; the tricarboxylic acid cycle (TCA cycle); starch and sucrose metabolism; and sphingolipid metabolism (Figure 1E).

**Figure 2:**
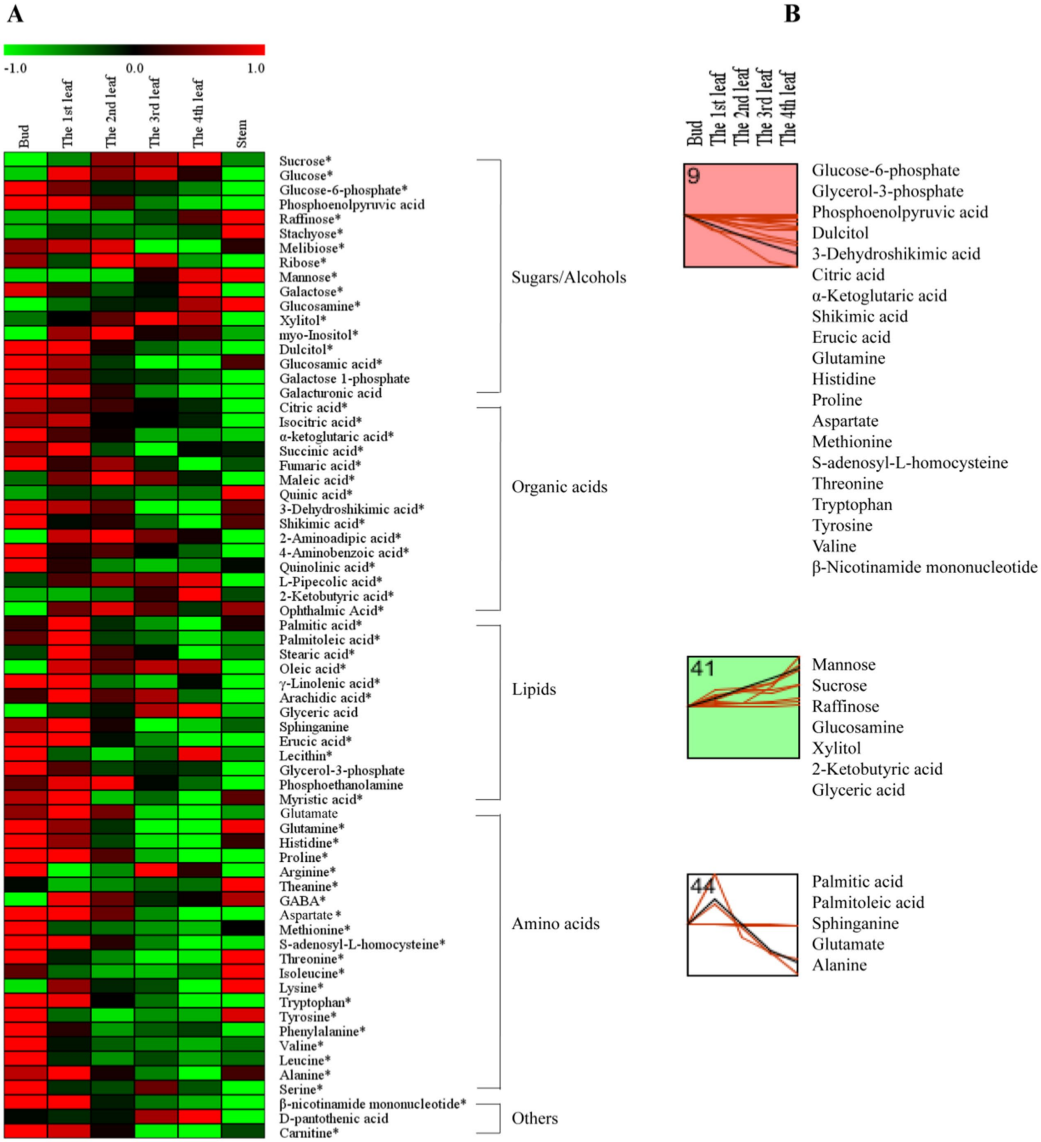
The primary metabolite changes based on comprehensive analysis in different tissues. **(A)** Heatmap of levels of differential primary metabolites in the bud, 1st-4th leaves, and stem. **(B)** *k*-means clustering analysis of differential primary metabolite levels in the bud and 1st-4th leaf. The number on the top left-hand corner of a profile box is the *k*-means profile ID number. *Compounds verified by authentic standards. Three biological replicates were analyzed for each group.

To further investigate the accumulation patterns of the 68 differential primary metabolites among different tissues, a comprehensive heatmap analysis and *k*-means clustering were performed (Figure 2). Red indicates that the primary metabolite level was higher than the mean level, while green indicates that the primary metabolite was at a lower level (Figure 2A). Four different profiles were clustered as shown in Figure 2B.

Sugars are the main products of photosynthesis in plants and are the most abundant biomolecules on earth, playing a variety of roles, such as energy and carbon transport molecules, and hormone-like signaling factors, etc. (19). In this study, the concentrations of mannose, raffinose, glucosamine, and xylitol increased gradually from the bud to the 4th leaf (Figure 2B, Profile 41); in contrast, the dulcitol concentration decreased persistently with increasing leaf maturity (Figure 2, Profile 9). As hexose monosaccharides, mannose, galactose and glucose are epimers (20), and among them, mannose is a component in the primary cell wall (21); the concentration of mannose markedly increased with increasing leaf maturity (from the bud to the 4th leaf) (Figure 2B, Profile 41). Raffinose (a trisaccharide) and subsequent higher molecular weight oligosaccharides (stachyose, etc.) are synthesized from dulcitol (i.e., galactinol) and are closely related to plant growth and development, adversity stress, etc. (22). As shown in Figure 2B, raffinose and dulcitol exhibited opposite accumulation trends with increasing leaf maturity; raffinose showed the highest level in the 4th leaf, and dulcitol showed the highest level in the bud. Xylitol is a pentose alcohol that is an ideal sweetener for patients with diabetes (23). In fresh tea leaves, the highest level of xylitol was observed in the 4th leaf (Figure 2B, Profile 41).

Organic acids play important roles in the metabolism of plants. They are not only early products of photosynthesis and respiration but also precursors for synthesizing other compounds (24). Our results showed that the levels of the organic acids associated with respiration, such as glucose-6-phosphate (involved in glycolysis) and citric acid, isocitric acid, α-ketoglutaric acid, succinic acid, and fumaric acid (involved in the tricarboxylic acid (TCA) cycle), were generally higher in young leaves (Figure 2A). Our results were consistent with those of a previous study (25). In addition, the contents of 3-dehydroshikimic acid and shikimic acid persistently decreased with increasing leaf maturity (Figure 2B, Profile 9), while the 2-ketobutyric acid content persistently increased (Figure 2B, Profile 41). 2-Ketobutyric acid is a substance involved in amino acids (glycine, methionine, valine, leucine, serine, threonine, and isoleucine) metabolism and is also among the degradation products of threonine (16).

Lipids are the main components of plant tissues, serving as fundamental constituents of cell membranes and playing an essential role in energy storage and signaling; fatty acids are the most abundant class of plant lipids and exist in unsaturated or saturated forms (26). In this study, fatty acids of palmitic acid, palmitoleic acid, sphinganine, stearic acid, oleic acid, γ-linolenic acid, and arachidic acid in the 1st leaf were higher than those in the bud, 2nd, 3rd, and 4th leaves (Figure 2A). This is possibly because higher contents of fatty acids are needed to synthesize membrane phospholipids for the generation of new cell membranes during the rapid growth period of leaves (27). On the other hand, the erucic acid content persistently decreased with increasing leaf maturity (Figure 2B, Profile 9), and the glyceric acid content persistently increased with increasing leaf maturity (Figure 2B, Profile 41). Regarding the aforementioned metabolites, erucic acid is involved in the biosynthesis of unsaturated fatty acids, and palmitic acid and palmitoleic acid are associated with cutin, suberine and wax biosynthesis (Figure 1E).

Amino acids are the units of protein synthesis and also the products of protein degradation. Their dynamic changes indicate the status of nitrogen nutrient metabolism in plants. Amino acids account for 2–4% of dry tea weight and are closely related to the taste and aroma of teas (16). As shown in Figure 2A, the levels of glutamine, histidine, proline, aspartate, methionine, s-adenosyl-l-homocysteine, threonine, tryptophan, tyrosine, and valine decreased with increasing leaf maturity (from the bud to the 4th leaf) (Figure 2B, Profile 9). Glutamate and alanine levels increased in the 1st leaf compared with the bud and then decreased with increasing leaf maturity (Figure 2B, Profile 44). In addition, the theanine content decreased from 14.14 mg/g in the bud to 11.19 mg/g in the 1st leaf and 11.72 mg/g in the 2nd leaf and then increased to 12.72 mg/g in the 3rd leaf and 12.52 mg/g in the 4th leaf; in particular, theanine accumulation was greater in the stem (28.12 mg/g) compared with that in the bud and leaves (Table 2). This trend is consistent with those observed in previous studies (13, 14). This is likely because theanine is mainly synthesized in the tea root and then transported to new nutritional tissues *via* the stem (28, 29).

**Table 2:** Quantitative analysis of theanine, alkaloids and flavanols in JHKC tea plant tissues by UPLC-PDA-TQS (mg/g).

Compounds	Bud	1st leaf	2nd leaf	3rd leaf	4th leaf	Stem
Theanine	14.14 ± 0.17 b	11.19 ± 0.47 d	11.72 ± 0.30 d	12.72 ± 0.26 c	12.52 ± 0.65 c	28.12 ± 0.10 a
Theobromine	7.82 ± 0.22 a	4.74 ± 0.25 b	2.87 ± 0.02 c	1.92 ± 0.04 d	1.08 ± 0.11 f	1.44 ± 0.02 e
Caffeine	37.41 ± 0.44 b	39.17 ± 0.82 a	35.69 ± 0.54 c	32.12 ± 0.84 d	27.96 ± 1.77 e	21.23 ± 0.23 f
Theacrine	14.68 ± 0.47 d	15.27 ± 0.82 cd	15.33 ± 0.26 cd	17.26 ± 0.64 b	19.45 ± 1.01 a	16.08 ± 0.33 c
Total alkaloids	59.91 ± 1.02 a	59.18 ± 1.79 a	53.88 ± 0.51 b	51.30 ± 1.52 bc	48.49 ± 2.87 c	38.75 ± 0.58 d
EC	5.55 ± 0.15 f	8.49 ± 0.31 d	10.25 ± 0.15 b	9.59 ± 0.28 c	7.70 ± 0.51 e	20.02 ± 0.41 a
ECG	33.51 ± 0.69 a	32.40 ± 0.41 ab	32.13 ± 0.73 ab	31.41 ± 0.87 bc	30.47 ± 1.38 c	16.10 ± 0.32 d
EGC	9.12 ± 0.12 a	20.50 ± 0.64 d	26.31 ± 0.23 b	26.06 ± 0.57 b	21.88 ± 1.18 c	31.25 ± 0.24 a
EGCG	58.57 ± 1.29 b	61.46 ± 0.83 a	56.31 ± 1.10 b	56.55 ± 2.14 b	56.94 ± 2.54 b	33.20 ± 0.80 c
C	5.78 ± 0.07 d	7.41 ± 0.36 c	8.82 ± 0.10 b	9.02 ± 0.44 ab	8.43 ± 0.49 b	9.54 ± 0.19 a
CG	ND	ND	ND	ND	ND	ND
GC	9.02 ± 0.13 d	12.86 ± 0.38 c	16.44 ± 0.31 b	19.29 ± 0.54 a	20.07 ± 1.28 a	15.90 ± 0.36 b
GCG	12.04 ± 0.14 a	12.68 ± 0.33 a	12.39 ± 0.33 a	12.11 ± 0.64 a	12.81 ± 0.78 a	6.54 ± 0.03 b
EGCG3”Me	0.01 ± 0.00 d	0.02 ± 0.00 c	0.02 ± 0.00 b	0.03 ± 0.00 a	0.03 ± 0.00 a	ND
Total catechins	133.60 ± 2.08 c	155.80 ± 2.68 b	162.67 ± 2.27ab	164.05 ± 5.32 a	158.32 ± 8.07ab	132.53 ± 0.63 c

The data represent the mean ± SD, *n* = 3. ND, not detected. Different letters indicate significant differences among the six groups according to one-way ANOVA (Tukey’s multiple comparison) (*p* < 0.05).

### 3.2. Secondary metabolite profiles among different tissues in JHKC

To comprehensively study the differential secondary metabolites among tissues of JHKC, the compounds were measured by metabolomics analysis. As shown in Figure 1B, QC samples were clustered at the center of the PCA score plot, demonstrating the stability of the LC–MS system. Similar to the primary metabolite profiles among different JHKC tissues, the secondary metabolite profiles of six tissues also showed very good separation, and the stem samples were at the edge of the PCA score plot. The HCA result also suggested that the stem exhibited the most distinct secondary metabolite pattern among the six tissues (Supplementary Figure S2B). Then, a PLS-DA model was constructed for the bud and leaf samples (Figure 1D). The bud, 1st-4th leaves showed significant differences in the PLS-DA score plot. The cross validation demonstrated that the *R^2^* and *Q^2^* intercepts were 0.54 and −0.44, respectively, indicating that the PLS-DA model was reliable (Supplementary Figure S2D).

A total of 51 secondary metabolites (Table 3) showed significant differences among the bud and 1st-4th leaves (VIP > 1; *p* < 0.05, Duncan’s test). These compounds included eight alkaloids, 13 flavanols, nine dimeric flavanols, 19 flavonol/flavone glycosides, and two other compounds.

**Table 3:** Summary of putatively identified secondary metabolites in JHKC.

RT (min)	Accurate mass (m/z)	Compound identification	MS^2^ fragments	*P*-value among 5 groups	VIP
Alkaloids					
5.01	167.0564	7-Methylxanthine*	167, 124, 150	0.007	1.55
7	181.0722	Theobromine*	138, 110, 83	0	9.78
9.64	195.0879	Caffeine*	138, 110, 69	0	4.75
6.56	211.0827	1,3,7-Trimethyluric acid*	211, 196, 154	0	3.25
8.95	225.0985	Theacrine*	225, 168, 197	0	6.01
1.53	104.1071	Choline*	104, 60	0.015	2.51
1.6	184.0734	Phosphocholine*	146, 129, 118	0	2.52
8.11	298.097	5′-Methylthioadenosine*	136, 298	0	1.69
Flavanols					
8.35	291.0866	C*	139, 123, 95	0	3.64
11.94	443.0973	CG*	153, 287	0	2.14
6.16	307.0815	GC*	223, 195, 163, 139	0	6.81
10.08	459.0927	GCG*	289, 181, 153, 139	0	3.51
9.95	291.0867	EC*	207, 139, 123, 55	0	7.08
11.27	443.0978	ECG*	273, 153, 139, 123	0.007	2.96
8.17	307.0816	EGC*	289, 153, 139	0	12
9.28	459.0926	EGCG*	441, 289, 153, 139	0.017	1.64
10.6	473.1082	EGCG3″Me*	139, 121, 167	0	3.73
11.42	275.0915	Epiafzelechin*	191, 139, 107, 55	0	2.77
12.98	427.1025	Epiafzelechin 3-gallate*	275, 153, 139, 107	0.006	1.62
12.82	453.118	EGC 3-coumaroate	119, 147, 289	0	1.43
7.56	453.1393	EGC 3-glucoside	307, 153, 139	0	1.92
Dimeric flavanols					
8.05	579.15	Procyanidin B1*	409, 291, 289, 127	0	2.48
8.64	579.1498	Procyanidin B2*	409, 301, 289, 127	0.004	1.05
7.34	579.1498	Procyanidin B3*	409, 271, 127	0	1.01
7.97	915.1616	Theasinensin A	897, 763, 139, 153	0	2.26
7.22	763.151	Theasinensin B	595, 443, 305v139	0	2.21
8.8	899.1667	Theasinensin F	425, 287, 153	0	2.87
8.74	731.1609	EC-(4alpha- > 8)-ECG	657, 265, 355	0.044	1.57
7.05	595.1447	EC-(4beta- > 8)-EGC	595, 485, 577, 469	0.044	3.03
6.02	595.1446	GC-(4alpha- > 8)-EC	595, 469, 577	0.002	2.31
Flavonol glycosides and flavone glycosides					
16.62	449.1081	Kaempferol-3-glucoside*	287, 85	0	8.11
16.64	595.1661	Kaempferol-3-rutinoside*	449, 287, 147, 331	0	5.14
15.85	757.2189	Kaempferol 3-glucosylrutinoside	595, 449, 287, 331	0.006	4.41
19.19	595.1447	Kaempferol-3-coumaroyglucoside	287, 147, 91, 311	0	1.24
20.12	741.1813	Kaempferol 3-dicoumarylgalactoside	449, 287	0	4.47
20.22	741.1813	Kaempferol 3-dicoumarylglucoside	455, 287, 147, 477	0	2.79
15.55	601.1186	Kaempferol 3-(6″-galloylglucoside)	287, 153, 125	0	1.17
14.62	465.1029	Quercetin-3-galactoside*	303, 165, 91	0	2.06
14.84	465.103	Quercetin-3-glucoside*	325, 185, 85, 55	0	8.05
14.78	611.161	Quercetin 3-rutinoside*	465, 303, 85	0	4.93
14.07	773.2135	Quercetin 3-glucosylrutinoside	611, 465, 303	0.023	3.61
13.89	479.082	Quercetin 3-glucuronide	301, 151, 178	0	1.85
12.91	481.0979	Myricetin 3-glucoside*	319	0	3.37
12.81	481.0979	Myricetin 3-galactoside*	319, 127, 85	0	1.92
14.17	433.1128	Isovitexin*	313, 283, 121, 81	0	1.92
13.34	433.113	Vitexin*	313, 139, 85	0	1.73
10.87	595.1657	Apigenin-6,8-C-diglucoside*	559, 475, 307, 153	0	3.15
12.12	565.1553	Apigenin-6-C-glucosyl-8-C-arabinoside	427, 409, 391, 379	0	4.64
12.38	565.1553	Apigenin-6-C-arabinoside-8-C-glucoside*	481, 427, 409, 325	0	3.86
Others					
19.98	471.2206	Linalyl primeveroside*	335, 333	0	1.62
2.74	337.1606	Theanine glucoside*	301, 208, 158	0	2.09

*The compound was identified by authentic compound (Metabolomics Standard Initiatives level 1); other compounds were identified by comparing their MS^2^ fragments with MassBank and HMDB databases (Metabolomics Standard Initiatives level 2); 5 groups were the bud and 1st-4th leaves.

Secondary metabolites are closely related to agronomic traits, yield, flavor quality, and resistance in tea trees (16). To understand the differences in the metabolic pathways among the bud and leaf samples, metabolic pathway analysis of the 51 differential secondary metabolites was performed by MetaboAnalyst 4.0 (Figure 1E). The results showed that the flavone and flavonol biosynthesis, caffeine metabolism, and flavonoid biosynthesis pathways differed significantly among tea leaves of different maturities.

**Figure 3:**
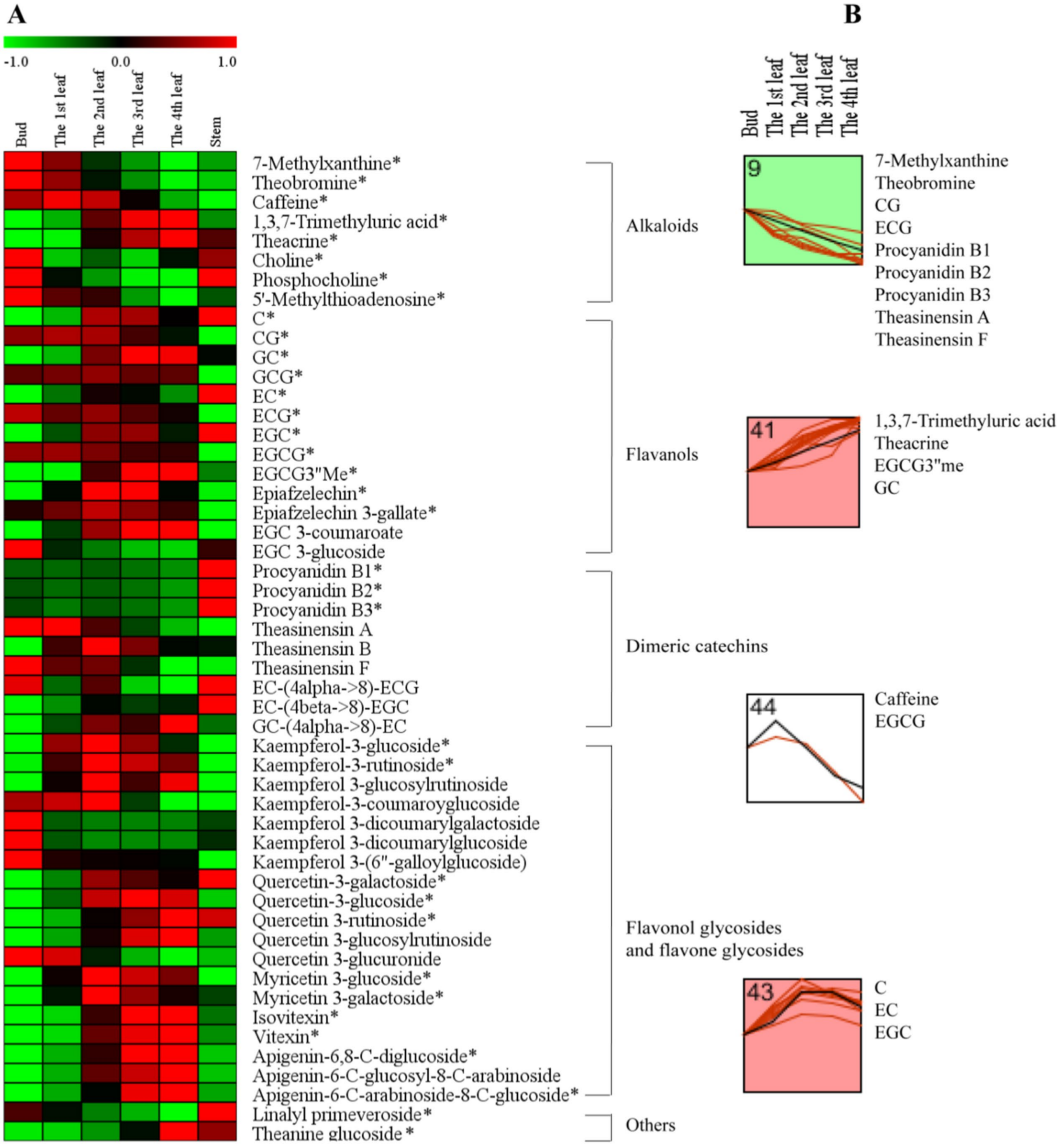
The secondary metabolite changes of comprehensive analysis in different tissues. **(A)** Heatmap of levels of differential secondary metabolites in the bud, 1st-4th leaves, and stem. **(B)** *k*-means clustering analysis of differential secondary metabolite levels in the bud and 1st-4th leaves. The number on the top left-hand corner of a profile box is the *k*-means profile ID number. *Compounds verified by authentic standards. Three biological replicates were analyzed for each group.

To further understand the accumulation patterns of the 51 differential secondary metabolites among different tissues, we conducted a network analysis of heatmap and *k*-means clustering algorithm analysis (Figure 3). A red color indicates that the secondary metabolite level was higher, while a green color indicates that the content of a secondary metabolite was lower (Figure 3A). Four different profiles were clustered as shown in Figure 3B.

Alkaloids are important bioactive and bitter components in tea, and their contents generally decrease with increasing leaf maturity (13, 15, 16, 25). In this study, caffeine, theacrine and theobromine were the dominant purine alkaloid components in JHKC (Table 2), which was consistent with the results of our previous study (1). The level of total alkaloids (theobromine, caffeine and theacrine) decreased from the bud to the 4th leaf (Table 2); however, different trends were observed for the changes of caffeine, theacrine and theobromine (Figure 3B). The trend of theobromine content was identical to that in the literature (13, 15, 16, 25), which decreased with tea leaf maturity (Figure 3B, Profile 9). The concentration of caffeine increased from the bud to the 1st leaf and then decreased from the 2nd leaf to the 4th leaf in JHKC (Figure 3B, Profile 44; Table 2). The 1st leaf showed the highest content of caffeine (39.17 ± 0.82 mg/g), and this distribution pattern of caffeine in JHKC was slightly different with that in the bud of tea plants *Camellia sinensis* var. *sinensis* cv. Longjing 43 and *Camellia sinensis var. sinensis* cv. Tieguanyin exhibited the highest caffeine content in previous studies (13, 25). Interestingly, the theacrine concentration increased with increasing leaf maturity (from the bud to the 4th leaf) (Figure 3B, Profile 41) and was the highest (19.45 ± 1.01 mg/g) in the 4th leaf (Table 2). This distribution pattern of theacrine in the JHKC was consistent with previous observations (30, 31) and differed with that in Yunnan Kucha tea [*Camellia kucha* (Chang et Wang) Chang] (15, 30, 32).

Flavanols, including non-epimeric flavanols (C, GC, CG, and GCG) and epiform flavanols (EC, EGC, ECG, and EGCG), are primary beneficial compounds for health in teas (16). In this study, four different profiles of flavanols were observed among the five tissues (from bud to the 4th leaf) (Figure 3B). The CG and ECG concentrations markedly decreased with increasing leaf maturity (from the bud to the 4th leaf) (Figure 3B, Profile 9). The ECG content in the bud was higher than that in the 1st-4th leaves, which agrees well with previous findings (2, 13, 25, 30). The EGCG3”Me and GC contents significantly increased with increasing leaf maturity (Figure 3B, Profile 41). Jin et al. reported that the EGCG3”Me content shows an increasing trend with leaf maturity to a certain extent (30), which agrees with our conclusion. In contrast, the GC content completely differed from that of previous studies (2, 25), which indicated that the GC content was higher in the bud than the leaves. The highest level of EGCG was observed among the flavanols in JHKC (Table 2), as observed in a similar accumulation pattern trend as caffeine. Its concentration was higher in the 1st leaf when compared with that in the bud and then decreased from the 2nd leaf to the 4th leaf (Figure 3B, Profile 44; Table 2); thus, the 1st leaf showed the highest EGCG content (61.46 ± 0.83 mg/g). Similar to caffeine, the above result also did not completely agree with the EGCG distribution pattern in other tea plants, including *Camellia sinensis* var. *sinensis* cv. Longjing 43 and *Camellia sinensis var. sinensis* cv. Tieguanyin, in which the bud showed the highest EGCG content (13, 25). The contents of C, EC, and EGC increased from the bud to the 2nd leaf, remained stable in the 3rd leaf, and then decreased in the 4th leaf (Figure 3B, Profile 43). Sun et al. (25) elucidated that the content of C in the bud (tea plants of *Camellia sinensis var. sinensis* cv. Longjing 43) were higher than those in the 1st-4th leaves; in addition, the contents of EC and EGC were higher in the 1st leaf than in the bud and decreased with increasing leaf maturity. This finding does not correspond with our results, possibly due to the different varieties used. Overall, our results demonstrated that nongallated flavanols (C, GC, EC, and EGC) contents increased while gallated flavanols (CG, ECG, and EGCG) contents decreased with increasing leaf maturity in JHKC (Figure 3A).

Procyanidins and theasinensins are important polymeric flavanols in tea. The contents of procyanidins (procyanidin B1, B2 and B3) and theasinensins (theasinensin A and F) were significantly decreased with increasing leaf maturity (from the bud to the 4th leaf) (Figure 3B, Profile 9); however, the stem showed the highest procyanidin contents when compared with those in the bud and leaves (Figure 3A). These phenomena were also observed in a previous study (25) and were consistent with knowledge that a small amount of procyanidins accumulated in fresh tea leaves and a large amount of procyanidins were enriched in tea roots (33); however, whether procyanidins are synthesized in the root of tea plants remains unknown. Flavonol/flavone glycosides in tea are mainly divided into kaempferol glycoside, quercetin glycoside, myricetin glycoside, and apigenin glycoside according to the aglycone structure (25). Kaempferol-3-glucoside and kaempferol-3-rutinoside showed the highest contents in the 2nd leaf and then decreased with increasing tea maturity (Figure 3A); the contents were higher in the 1st leaf than the bud and decreased with increasing leaf maturity for the Longjing 43 variety (25). The contents of quercetin glycosides (quercetin-3-glucoside, quercetin-3-rutinoside and quercetin-3-glucosylrutinoside) and apigenin glycosides (isovitexin, vitexin, apigenin-6,8-C-diglucoside, apigenin-6-C-glucosyl-8-C-arabinoside and apigenin-6-C-arabinosid-8-C-glucoside) increased with leaf maturity (Figure 3A), which was consistent with the results of Sun’s study (25). In conclusion, the composition and distribution pattern of secondary metabolites varied in different cultivars and tissues in tea plants.

### 3.3. Analysis of an integrated primary/secondary metabolite network in different tissues

**Figure 4:**
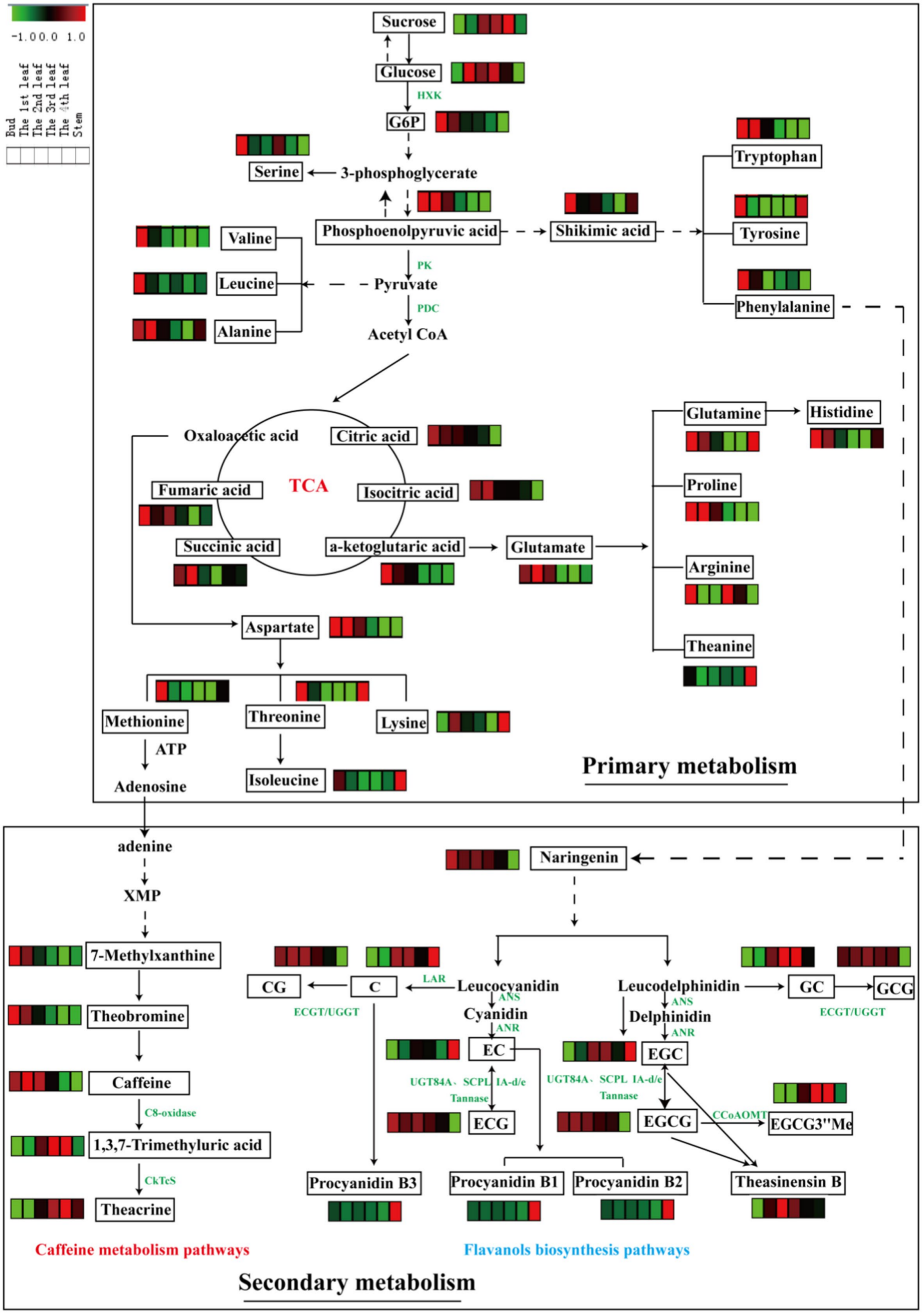
Metabolic pathways of differential primary and secondary metabolites in six tissues (bud, 1st-4th leaves, and stem). Three biological replicates were analyzed for each group.

To clarify the differences in metabolic pathways in tea leaves of different positions from a biochemical perspective, the distribution patterns of pivotal primary metabolites of sugars, organic acids and amino acids, as well as secondary metabolites of alkaloids and flavonoids, were mapped in their corresponding biosynthetic pathways (Figure 4).

#### 3.3.1. Glycolysis and TCA cycle

Glycolysis is a metabolic pathway that converts glucose into pyruvate; the pathway not only generates high-energy substrates (ATP and NADH) but also provides important precursors for various metabolic pathways, such as the TCA cycle and biosynthesis of secondary metabolites (34). In this study, the content of sucrose increased with leaf maturity, and glucose slightly accumulated in mature leaves relative to the bud (Figure 4). Glucose-6-phosphate and phosphoenolpyruvic acid, as intermediates of glycolysis, were enriched in the bud and 1st leaf and decreased gradually with leaf maturity. In general, photosynthesis increases with increasing leaf maturity, whereas respiration shows the opposite trend (10). This finding is consistent with our above results. The TCA cycle is an epicenter in cell metabolism, as many substrates can feed into the TCA cycle, which produces power and carbon skeletons (34). Pyruvate is converted to acetyl-CoA by the pyruvate dehydrogenase complex (PDC), and then acetyl-CoA serves as fuel for the TCA cycle. In this study, the accumulation patterns of the TCA metabolites were similar with the glycolysis intermediates that they were decreased with increasing leaf maturity (Figure 4).

#### 3.3.2. Amino acid metabolism

Amino acids are major transportable nitrogenous compounds in tea plants and contribute to the umami taste of tea. The biosynthesis of amino acids occurs primarily through the following pathways: glycolysis and TCA cycle. For example, phosphoenolpyruvic acid is the precursor of aromatic amino acids (tryptophan, tyrosine and phenylalanine) *via* the shikimic acid pathway; pyruvate is the precursor in the biosynthesis of valine, leucine and alanine; and α-ketoglutaric acid and oxaloacetic acid (TCA cycle intermediates) are the precursors of glutamate and aspartate family amino acids, respectively (10). Furthermore, glutamate and aspartate serve as precursors for the biosynthesis of many other amino acids, e.g., glutamine, proline, histidine, arginine and theanine (from glutamate) and methionine, lysine, threonine, and isoleucine (from aspartate). From a holistic perspective, the contents of the abovementioned amino acids were largely higher in the bud than in the 1st-4th leaves and stems except for theanine. Parallel phenomena were observed in a previous study (25).

#### 3.3.3. Caffeine metabolism

Caffeine metabolism is the main pathway to generate purine alkaloids in tea plants, and theacrine is a characteristic component of purine alkaloids in JHKC. As shown in Figure 4, adenine (downstream of methionine) is the most direct and effective precursor substance for the synthesis of tea purine alkaloids (35), which is subsequently converted to 7-methylxanthine and then theobromine, and theobromine is the precursor of caffeine. Theacrine is biosynthesized first through the conversion of caffeine to 1,3,7-trimethyluric acid by C8-oxidase and then converted to theacrine by N9-methyltransferase (CkTcS) (1). In our study, the accumulation rate of 7-methylxanthine, caffeine and theobromine was relatively higher in tender leaves; however, 1,3,7-trimethyluric acid and theacrine were comparatively higher levels in mature leaves. The C8-oxidase enzyme activity may significantly increase with leaf maturity. The higher content of theacrine in mature leaves of JHKC was possibly caused by the massive accumulation of the 1,3,7-trimethyluric acid intermediate in the later stage of leaf growth, which is a precursor to synthesize theacrine by CkTcS.

#### 3.3.4. Flavanols metabolism

Flavanols are important secondary metabolites derived from the phenylpropanoid pathway, and phenylalanine is the starting compound (34). A flavanols pathway was mapped in Figure 4, and anthocyanidin synthase (ANS), leucoanthocyanidin reductase (LAR), anthocyanin reductase (ANR), epicatechin: 1-O-galloyl-β-D-glucose O-galloyltransferase (ECGT) and UDP-glucose:galloyl-1-O-β-D-glucosyltransferase (UGGT) are key enzymes involved in the flavanols biosynthesis pathway. ANS catalyzes the conversion of leucocyanidin/leucodelphindin to cyanidin/delphinidin, LAR catalyzes the conversion of leucocyanidin/leucodelphindin to C/GC, and ANR catalyzes the conversion of cyanidin/delphinidin to EC/EGC (1). Furthermore, ECGT and UGGT are associated with the biosynthesis of galloylated flavanols (CG and GCG) (36).

In our study, non-galloylated flavanols (C and GC) were relatively lower in tender leaves; in contrast, galloylated flavanols (CG and GCG) were relatively higher, indicating that ECGT/UGGT may be involved. Moreover, other galloylated flavanols (ECG and EGCG) contents significantly decreased with the development of fresh leaves, according to the latest report that nongallated catechins (EC and EGC) are the precursors for the synthesis of gallated catechins (ECG and EGCG) by UGT84A and SCPL IA-d/e and are also the products of gallated catechins (ECG and EGCG) hydrolysis by a Tannase (37) (Figure 4). Therefore, the trends observed for the galloylated flavanols (ECG and EGCG) with leaf maturity are likely caused by the decreasing in synthesis enzymes (UGT84A and SCPL IA-d/e) activities with increasing leaf maturity and increasing activities by the hydrolysis enzyme (Tannase). The EGCG3”Me content markedly increased with increasing leaf maturity (from the bud to the 4th leaf) and is derived from EGCG *via* caffeoyl-CoA 3-O-methyltransferase (CCoAOMT). Procyanidin B1 [EC-(4b,8)-C] and procyanidin B2 [EC-(4b,8)-EC] are EC-based PAs, and procyanidin B3 [C-(4b,8)-C] is a C-based PAs (1). Interestingly, the procyanidins (B1, B2, and B3) displayed the highest level in the stem compared to other leaves, and an explanation is provided in paragraph 3.2 of this paper.

#### 3.3.5. Correlation analysis of primary metabolites and important secondary metabolites

**Figure 5:**
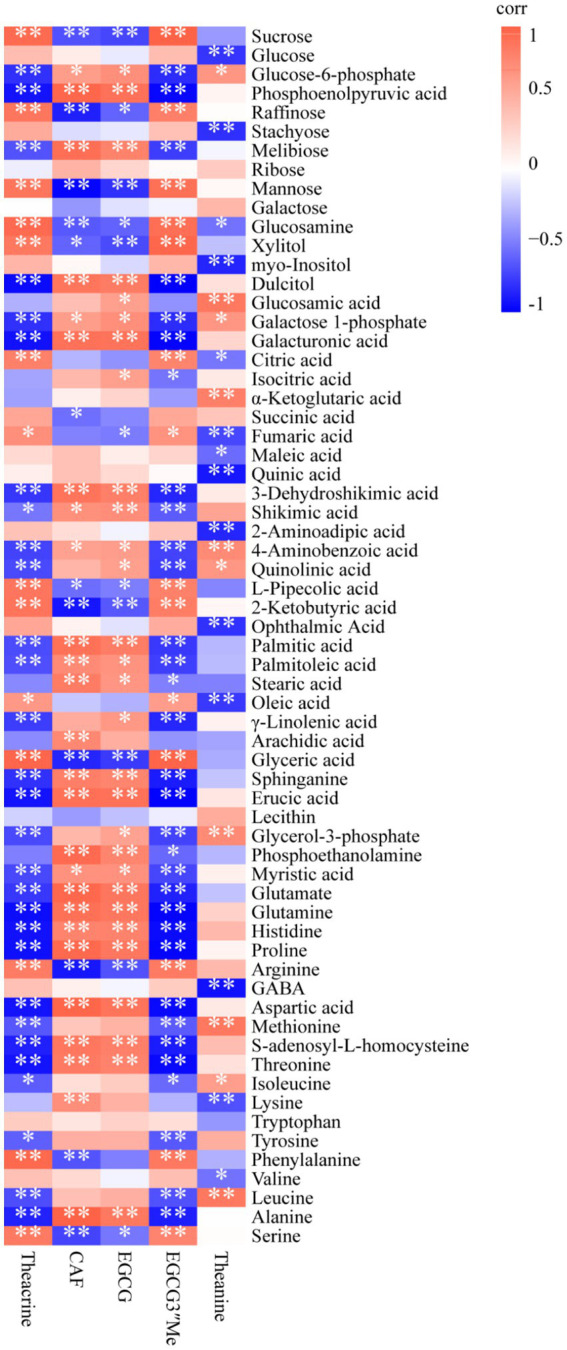
Heatmap of the correlation between primary metabolites and important specialized secondary metabolites of JHKC. The orange and blue colors represent positive and negative correlations, respectively. *p < 0.05; **p < 0.01.

To clarify the correlations between primary metabolites and important secondary metabolites (theanine, caffeine, EGCG, and theacrine), we performed a correlation analysis (Figure 5). In this figure, caffeine and EGCG showed similar correlations with primary metabolites, as did theacrine and EGCG3”Me. For example, sucrose was extremely significantly negatively correlated with caffeine (correlation corr = −0.68, *p* < 0.01) and EGCG (corr = −0.72, p < 0.01), while it was extremely significantly positively correlated with theacrine (corr = 0.85, p < 0.01) and EGCG3”Me (corr = 0.90, p < 0.01). Methionine is a precursor substance of the S-adenosyl-L-methionine cycle (SAM cycle), which is crucial for the synthesis of purine alkaloids in tea plants (35). It was highly significantly negatively correlated with theacrine (corr = −0.66, p < 0.01) but showed no significant correlation with caffeine (corr = 0.30, *p* > 0.05). EGCG and EGCG3”Me are important specialized secondary metabolites derived from the phenylpropanoid pathway, in which the primary metabolite phenylalanine is the starting compound (34). In this study, phenylalanine was nonsignificantly negatively associated with EGCG (corr = −0.51, p > 0.05) but was extremely significantly positively associated with EGCG3”Me (corr = 0.77, p < 0.01). Theanine, a specialized amino acid in tea, was negatively correlated with sucrose and glucose (corr = −0.41 and corr = −0.78, respectively), which might explain that sugar (glucose or sucrose) is the probable signaling molecule to regulate the enzymes expression in theanine metabolism (29).

Interestingly, the 2nd-4th leaves showed a weaker primary metabolism but stronger secondary metabolism comparing to bud and the 1st leaf (Figures 2, 3), this is possibly because of that the new tea shoots need more primary metabolites such as sugars, proteins, amino acids and lipids which are essential for life activities in growth and development period, while the mature leaves need more secondary metabolites in response to the surrounding environment (16).

## 4. Conclusion

In summary, our study provided insight into the chemical constituents at the interface of primary and secondary metabolism in JHKC tea plants by mass spectrometry-based platforms. The results showed that there were significant differences in 68 primary metabolites and 51 secondary metabolites in JHKC tea leaves with different maturities. The bud showed higher levels of primary metabolites, including sugars/alcohols (such as glucose-6-phosphate, phosphoenolpyruvic acid and dulcitol), organic acids (such as 3-dehydroshikimic acid, citric acid, α-ketoglutaric acid, and shikimic acid), lipids (such as erucic acid and glycerol-3-phosphate), amino acids (such as glutamine, histidine, proline, aspartate, methionine, s-adenosyl-l-homocysteine, threonine, tryptophan, tyrosine, and valine), and β-nicotinamide mononucleotide, as well as secondary metabolites of alkaloids (such as 7-methylxanthine and theobromine) and flavonoids (such as CG, ECG, procyanidin B1, procyanidin B2, procyanidin B3, theasinensin A, and theasinensin F). The 1st leaf presented higher levels of primary metabolites of lipids (such as palmitic acid, palmitoleic acid and sphinganine) and amino acids (glutamate and alanine) as well as secondary metabolites of caffeine and EGCG. The 4th leaf presented higher levels of primary metabolites of sugars/alcohols (such as mannose, sucrose, raffinose, glucosamine, and xylitol), 2-ketobutyric acid of organic acids, and glyceric acid of lipids and secondary metabolites of alkaloids (1,3,7-trimethyluric acid and theacrine) and flavonoids (EGCG3”Me and GC). Among them, caffeine, EGCG, and theacrine showed characteristics distribution patterns in JHKC when compared with *Camellia sinensis* var. *sinensis* cultivars and Yunnan Kucha tea [*Camellia kucha* (Chang et Wang) Chang]. This study provides a novel interpretation of compound spatial distribution from the perspectives of both primary and secondary metabolism and will be helpful for efficiently utilizing important specialized metabolites in JHKC tea plants.

## Data availability statement

The original contributions presented in the study are included in the article/Supplementary material, further inquiries can be directed to the corresponding authors.

## Author contributions

WW: conceptualization, methodology, formal analysis, investigation, writing–original draft, writing–review and editing, and funding acquisition. JS: methodology, formal analysis, investigation, writing–original draft, and writing–review and editing. JJ and ZheL: formal analysis. YY: investigation. ZC: resources. SZ: formal analysis and investigation. WD: conceptualization, formal analysis, writing–review and editing, and funding acquisition. ZhiL: conceptualization, writing–review and editing, project administration, and funding acquisition. All authors contributed to the article and approved the submitted version.

## Funding

This work was supported by the Hunan Provincial Natural Science Foundation of China (2021JC0007), the Hunan Agricultural Science and Technology Innovation Funds of China (2022CX33), the Key Laboratory Open Funds of Ministry of Agriculture and Rural Affairs of China (TZDZW202204), and the Foundation of Public Projects of Zhejiang Province (LR23C160002).

## Conflict of interest

Author YY was employed by Hunan Tea Group Co., Ltd. and ZC was employed by Chenzhou Guyanxiang Tea Co., Ltd.

The remaining authors declare that the research was conducted in the absence of any commercial or financial relationships that could be construed as a potential conflict of interest.

## Publisher’s note

All claims expressed in this article are solely those of the authors and do not necessarily represent those of their affiliated organizations, or those of the publisher, the editors and the reviewers. Any product that may be evaluated in this article, or claim that may be made by its manufacturer, is not guaranteed or endorsed by the publisher.

## Footnotes

^1^ www.r-project.org/

^2^ https://www.metaboanalyst.ca/

## Abbreviations

EGCG: epigallocatechin-3-gallate
EGC: epigallocatechin
ECG: epicatechin gallate
EC: epicatechin
GCG: gallocatechin gallate
GC: gallocatechin
CG: catechin gallate
C: catechin
EGCG3″Me: epigallocatechin-3-O-(3″-O-methyl)-gallate
Thea: theanine; TB, theobromine
CAF: caffeine
TC: theacrine
GABA: gamma aminobutyric acid
isovitexin: apigenin-6-C-glucoside
vitexin: apigenin-8-C-glucoside
UHPLC-QTOF/MS: ultra-high performance liquid chromatography quadrupole time-of-flight mass spectrometry
