# Supplementary material for: Comprehensive metabolic analyses provide new insights into primary and secondary metabolites in different tissues of Jianghua Kucha tea (Camellia sinensis var. assamica cv. Jianghua)
Source: Front Nutr. 2023 May 19;10:1181135. doi: 10.3389/fnut.2023.1181135 (PMC10235520; doi:10.3389/fnut.2023.1181135)
Supplement: Supplementary file 1 [file Data_Sheet_1.docx]

**Comprehensive metabolic analyses** [**provide new insights into**](https://onlinelibrary.wiley.com/doi/abs/10.1111/pce.12951)**primary and secondary metabolites** **in different tissues of Jianghua Kucha tea (*Camellia sinensis* var. *assamica* cv. Jianghua)**

**Wenliang Wu^1, 2, †^, Jiang Shi^1, †^, Jiqiang Jin^1^, Zhen Liu^2^, Yong Yuan^4^, Zhida Chen^3^, Shuguang Zhang^2^, Weidong Dai^1, *^ and Zhi Lin^1, *^**

^1^ Key Laboratory of Tea Biology and Resources Utilization, Ministry of Agriculture, Key Laboratory of Biology, Genetics and Breeding of Special Economic Animals and Plants, Ministry of Agriculture and Rural Affairs, Tea Research Institute, Chinese Academy of Agricultural Sciences, 9 Meiling South Road, Hangzhou, Zhejiang 310008, PR China

^2^ Tea Research Institute, Hunan Academy of Agricultural Sciences, Changsha, Hunan 410125, PR China

^3^ Chenzhou Guyanxiang Tea Co., Ltd., Chenzhou, Hunan 423022, PR China

^4^ Hunan Tea Group Co., Ltd., Changsha, Hunan 410126, PR China

**^†^**These authors have contributed equally to this work and share first authorship

* **Correspondence**: Weidong Dai, [daiweidong@tricaas.com](mailto:daiweidong@tricaas.com); Zhi Lin, linzhi@caas.cn.

***Purchase information of standards***

Flavanols of EGCG, EGC, ECG, EC, GCG, GC, CG, and C, 1,3,7-trimethyluric acid, kaempferol-3-glucoside, quercetin-3-glucoside, quercetin-3-galactoside, myricetin 3-galactoside, naringenin, glutamine, histidine, proline, arginine, theanine, GABA, aspartic acid, methionine, s-adenosyl-l-homocysteine, threonine, isoleucine, lysine, tryptophan,tyrosine, phenylalanine, valine, leucine, alanine, serine, theogallin, chlorogenic acid and 5-methoxysalicylic acid were obtained from Sigma (St. Louis, MO, USA). Theacrine, theobromine and caffeine were purchased from Yuanye (Shanghai, China). Procyanidin B1, procyanidin B2 and procyanidin B3 were gained from ChemFaces (Wuhan, Hubei, China). 2-Aminoadipic acid, 4-aminobenzoic acid, quinolinic acid, methionine sulfoximine, l-pipecolic acid, 2-ketobutyric acid, ophthalmic acid, epiafzelechin, and epiafzelechin 3-gallate were obtained from Mreda (Beijing, China). Sucrose, glucose, glucose-6-phosphate, raffinose, stachyose, melibiose, ribose, mannose, galactose, glucosamine, xylitol, myo-inositol, dulcitol, glucosamic acid, citric acid, isocitric acid, succinic acid, maleic acid, quinic acid, 3-dehydroshikimic acid, shikimic acid, palmitic acid, palmitoleic acid, stearic acid, oleic acid, γ-linolenic acid, arachidic acid, erucic acid, lecithin, myristic acid, β-nicotinamide mononucleotide, and carnitine were obtained from J&K Scientific Ltd. (Beijing, China). EGCG3″Me was purchased from Nagara Science Co., Ltd. (Gifu, Japan). Linalyl primeveroside was synthesized by the National Glycoengineering Research Center, Shandong University, Jinan, China.

***Raw data analysis***

Raw metabolomics data acquired from UHPLC−QTOF/MS were first treated for peak picking using MassHunter Qualitative Analysis software (B.07.00 SP1, Agilent Technologies, Santa Clara, CA, USA) and then for peak alignment using Mass Profiler Professional (version 13.0, Agilent Technologies, Santa Clara, CA, USA). The signal-to-noise (S/N) threshold for peak detection was set to 3. The retention time tolerance and mass tolerance for the peak alignment was set to 0.2 min and 0.01 Da, respectively. The obtained compound ion features with relative standard deviations (RSD) of mass intensities less than 30% in QC samples were used for multivariate and univariate analysis. Compounds were structurally identified based on authentic standards, MS^2^ spectra, mass accuracy, and metabolomics databases (Metlin and HMDB).

**
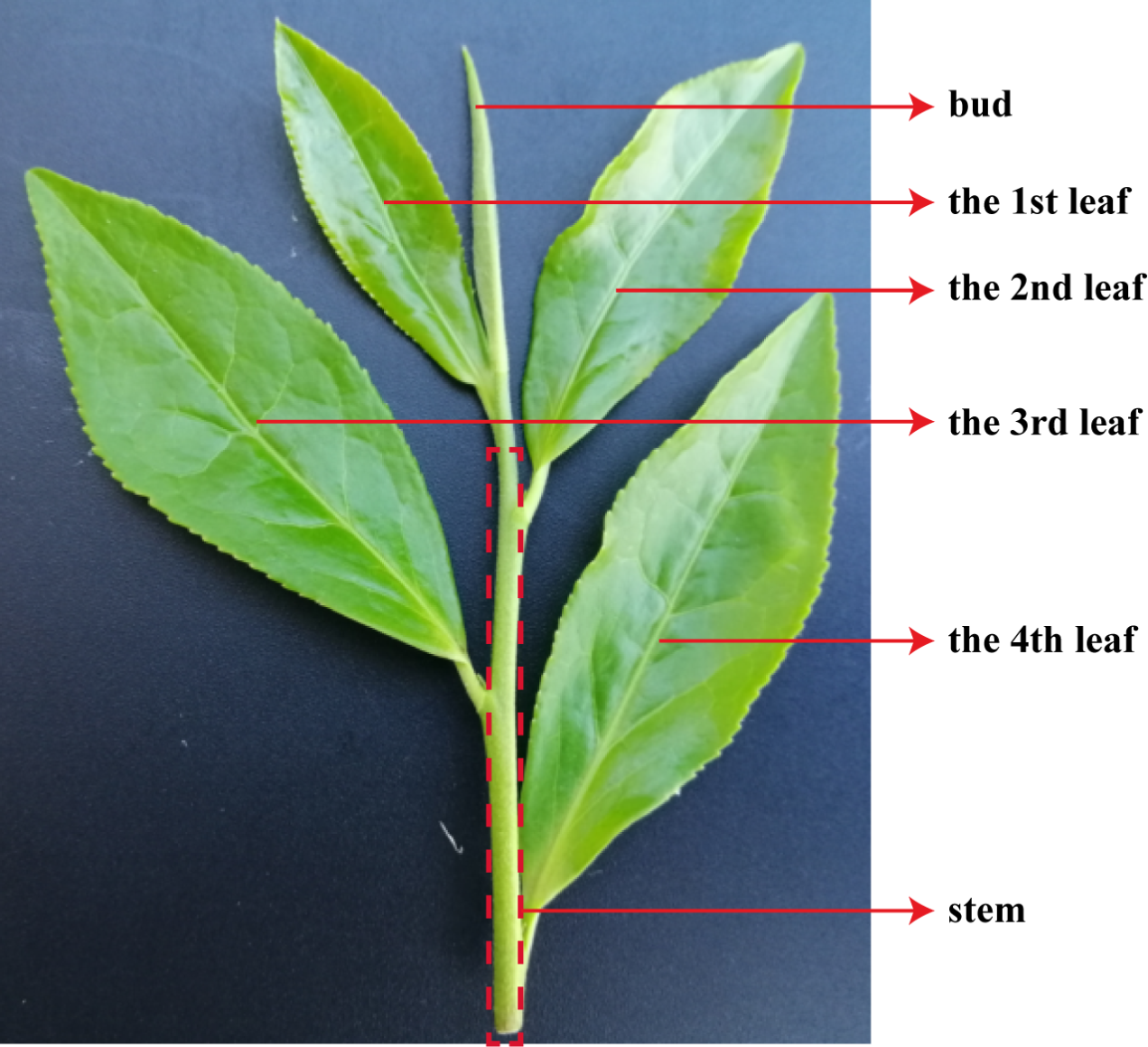
**

**Figure S1.** Photograph of the bud, 1st-4th leaves, and new stem of JHKC tea plant used for primary and secondary metabolite analysis.


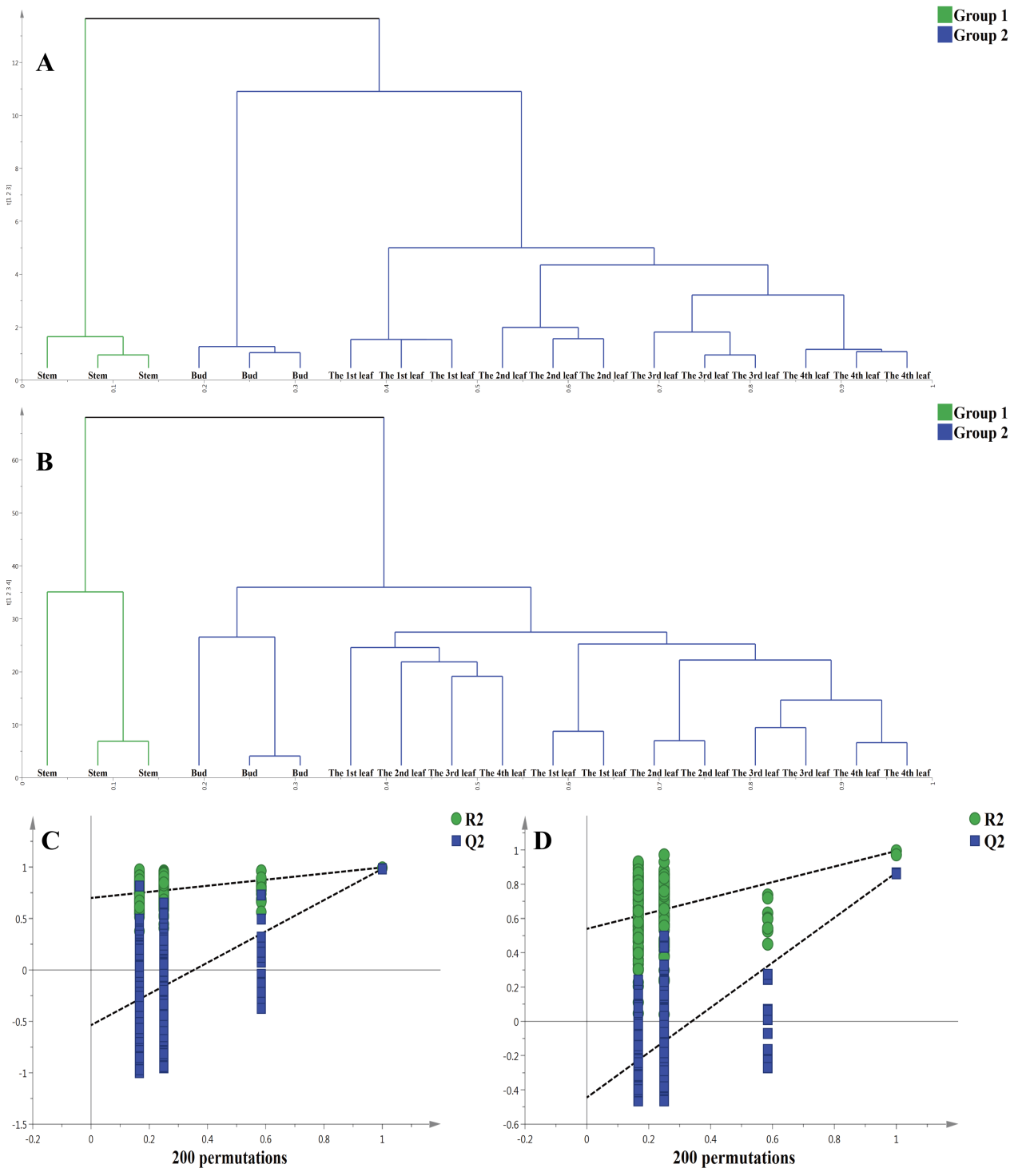


**Figure S2.** Hierarchical cluster analysis (HCA) of the tea samples of different maturity in JHKC based on primary metabolites (A) and secondary metabolites (B); cross-validation plot of PLS-DA model with 200 permutation tests based on primary metabolites (C): the intercepts of *R^2^* and *Q^2^* were 0.7 and -0.47, respectively, and based on secondary metabolites (D): the intercepts of *R^2^* and *Q^2^* were 0.54 and -0.44, respectively.
